# Unraveling the Length of Hospital Stay for Patients with Urinary Tract Infections: Contributing Factors and Microbial Susceptibility

**DOI:** 10.3390/antibiotics14040421

**Published:** 2025-04-21

**Authors:** Deema Rahme, Hania Nakkash Chmaisse, Pascale Salameh

**Affiliations:** 1 Ecole Doctorale des Sciences et des Technologies, Lebanese University, Hadat P.O. Box 6573/14, Lebanon; 2 Faculty of Pharmacy, Beirut Arab University, Beirut P.O. Box 11-5020, Lebanon; rahme@bau.edu.lb; 3 INSPECT-LB (Institut National de Santé Publique, d’Épidémiologie Clinique et de Toxicologie—Liban), Beirut P.O. Box 12109, Lebanon; salameh.p@unic.ac.cy; 4 Gilbert and Rose-Marie Chagoury School of Medicine, Lebanese American University, Byblos P.O. Box 36, Lebanon; 5 Department of Primary Care and Population Health, University of Nicosia Medical School, 2417 Nicosia, Cyprus

**Keywords:** length of stay, antimicrobial stewardship, drug resistance, inappropriate prescribing, Lebanon

## Abstract

**Background/Objectives:** Length of hospital stay (LOS) is a critical measure of healthcare efficiency. This study investigated factors contributing to prolonged LOS in adult patients with urinary tract infections (UTIs) in Lebanon and assessed microbial susceptibility patterns of causative pathogens. **Methods:** A retrospective cohort study was conducted across five Lebanese university hospitals (March 2022–December 2023), analyzing 401 patients. Data on microbiological findings and the LOS were extracted from medical records. Statistical analyses, including descriptive statistics, bivariate tests (*t*-tests, ANOVA, and Pearson’s correlation), and multiple linear regression (significance: *p* ≤ 0.05), were performed using IBM SPSS^®^ 27. **Results:** The mean LOS was 5.85 ± 2.41 days. Prolonged hospitalization was associated with patient-related factors (age, comorbidities, UTI type, specific symptoms, and multidrug-resistant infections) and treatment-related factors. Empirical use of carbapenems (β = 0.783, *p* = 0.004) and fluoroquinolones (β = 1.360, *p* = 0.014), along with inappropriate antibiotic prescriptions (β = 0.609, *p* = 0.022), significantly extended the LOS. Conversely, antibiotic de-escalation based on culture results reduced the LOS (β = −0.567, *p* = 0.029). *Escherichia coli* (61.8%) was the predominant pathogen, followed by *Klebsiella pneumoniae* (11.9%), *Proteus mirabilis* (7.8%), and *Pseudomonas aeruginosa* (7.5%). Notably, susceptibility to antibiotics showed a concerning decline. **Conclusions:** Inappropriate antibiotic prescriptions were linked to a prolonged LOS, emphasizing the need for judicious antimicrobial use. The positive impact of de-escalation supports culture-guided therapy. Declining antibiotic susceptibility highlights the urgency for robust antimicrobial stewardship programs (ASPs) and a national microbial resistance database to combat antimicrobial resistance (AMR) in Lebanon.

## 1. Introduction

Urinary tract infections (UTIs) are a common and significant cause of morbidity in both community and healthcare settings, with substantial implications for patient outcomes and healthcare systems [1,2]. Although UTIs can affect individuals of all ages and genders, certain populations, including the elderly, individuals with comorbidities, immunocompromised patients, and those with prolonged hospital stays, are at particularly high risk. In the inpatient setting, UTIs are frequently complicated by factors such as catheterization, antibiotic resistance, delayed diagnosis, or inappropriate treatment, all of which may contribute to an extended length of hospital stay (LOS) [3,4].

UTIs most commonly result from the ascending spread of uropathogens from the periurethral area to the bladder or kidneys [5]. The pathogenesis often involves bacterial adhesion to uroepithelial cells, followed by colonization, invasion, and, in catheter-associated urinary tract infections (CAUTIs), biofilm formation [6]. While bacteria are the primary causative agents, fungal pathogens, particularly *Candida* species, have emerged as important contributors, especially in immunosuppressed patients, those with indwelling urinary catheters, or those exposed to broad-spectrum antibiotics [5,7]. *Escherichia coli*, particularly uropathogenic strains (UPEC), remains the most common cause of both community-acquired and nosocomial UTIs worldwide, accounting for up to 90% and 50% of cases, respectively [8]. Other bacterial pathogens include *Klebsiella pneumoniae*, *Proteus mirabilis*, *Pseudomonas aeruginosa*, and *Enterococcus* species [9]. In Lebanon, *E. coli* continues to be the predominant uropathogen. A 10-year retrospective study in Beirut reported that *E. coli* accounted for 60.6% of urinary isolates, while a more recent investigation in South Lebanon found it responsible for 67.1% of cases [10,11]. These figures underscore its persistent clinical relevance in the local context. While limited data are available on fungal UTI prevalence in Lebanon, clinical observations suggest an increasing burden in hospitalized populations [12].

Antimicrobial resistance (AMR) presents an escalating challenge in the effective treatment of UTIs. The emergence of extended-spectrum-beta-lactamase (ESBL)-producing and carbapenem-resistant organisms has been documented in Lebanese hospitals, mirroring global and regional trends [11,13,14]. Although comprehensive national surveillance is lacking, available data from tertiary centers indicate rising resistance rates and frequent use of broad-spectrum empirical therapy [14,15]. This overuse, alongside insufficient ASPs and infection control practices, contributes to both higher rates of resistance and prolonged hospitalization [16,17,18]. The clinical impact of AMR on hospitalization is evident. In Lebanese patients, infections with resistant *E. coli* strains are associated with a longer median LOS (6 days) compared to infections with susceptible strains [19]. The effect is even more pronounced in cases involving device-associated infections, where the average LOS reaches 13.8 days [20]. The added complexity of managing fungal infections, often requiring longer or combined antifungal treatment, may further contribute to extended LOS in affected individuals [4,5]. LOS is a critical metric in healthcare delivery, used to evaluate the quality, efficiency, and cost-effectiveness of care [21,22,23]. A prolonged LOS is associated with increased healthcare costs, greater risk of hospital-acquired complications, and inefficient resource utilization [24,25]. Conversely, excessively short stays may risk premature discharge and higher readmission rates [24]. Understanding the factors contributing to LOS in UTI patients is thus essential for improving clinical outcomes and hospital performance.

International studies have identified key predictors of extended LOS, including advanced age, comorbidities, infection severity, and inappropriate or delayed antibiotic initiation [3,4,24]. However, these findings may not be fully generalizable to Lebanon, where local microbial patterns, resistance rates, healthcare infrastructure, and prescribing behaviors differ. Given these considerations, the current study aimed to address two primary objectives. First, it sought to investigate the clinical and microbiological factors associated with extended LOS among hospitalized UTI patients in Lebanon. Second, it aimed to assess the antimicrobial susceptibility patterns of isolated uropathogens.

## 2. Results

This study included 401 patients from various regions, with Beirut contributing the largest proportion (40.9%). The majority of cases (81%) were admitted to the internal medicine department.

### 2.1. Physician Characteristics

According to the data, 76.6% of the prescribing physicians were male. The majority of them (54.1%) were specialists, while 25.7% were attending physicians, 11% were consultants, and 9.2% were residents or fellows. The most common specialties among these physicians were internal medicine (50.4%), followed by infectious diseases (22.2%), urology/nephrology (11.2%), and critical care (10%). Only a small percentage (6.2%) specialized in other areas such as emergency medicine and general surgery.

### 2.2. Patient Characteristics

Patients had a mean age of 64.81 ± 16.88 years, with females constituting the majority (61.8%) and being married (71.1%). A considerable percentage of patients had medical insurance (58.4%) and were current smokers (33.9%). Chronic conditions were prevalent among patients, particularly hypertension (55.6%), dyslipidemia (40%), other cardiac conditions (35.7%), and diabetes (35.9%). In addition, a considerable proportion of patients (28.6%) had a history of urological conditions, some had neurological disorders (18.2%), and approximately one-fourth of them had a catheter inserted.

### 2.3. UTI Findings

As depicted in Table 1, pyelonephritis was the most prevalent form of UTI, comprising 47.4% of the cases, with complicated cystitis following closely at 42.6%. Patients commonly reported fever (51.1%), flank pain (40%), and dysuria (47.1%). Urine analysis and urine and blood cultures were typically employed in the diagnostic workup. The average temperature was 37.81 ± 0.94 °C, and the white blood cell count averaged 12.19 ± 5.09 cells/μL. The average length of hospital stay for these patients was 5.85 ± 2.41 days.

### 2.4. Microbiological Findings

The microbiological profile of the UTI patients revealed a high positive urine culture (88%). *Escherichia coli* was the predominant pathogen, isolated in 61.8% of cultures, followed by *Klebsiella pneumoniae* (11.9%), *Proteus mirabilis* (7.8%), and *Pseudomonas aeruginosa* (7.5%). ESBLs were detected in 37.7% of isolates, while 6.4% were MDROs. The antibiogram analysis revealed varied susceptibility patterns among Gram-negative bacteria. *E. coli* and *K. pneumoniae* showed the highest susceptibility to tigecycline, imipenem, and amikacin. *P. aeruginosa* was most susceptible to amikacin, gentamicin, and cefepime. Other *Enterobacterales* demonstrated high susceptibility to tigecycline, imipenem, and amikacin. For less common isolates, *Acinetobacter baumannii* was fully susceptible to colistin. *Enterococcus* spp. and *Coagulase-negative Staphylococcus* spp. showed high susceptibility to tigecycline and vancomycin. Overall, tigecycline, imipenem, and amikacin were the most effective antibiotics against the tested Gram-negative bacteria. These findings are presented in Table 2 and Table 3.

### 2.5. Antibiotic Prescriptions

Carbapenems were the most frequently prescribed antibiotics (43.9%), followed by 3rd-generation cephalosporins (27.7%). Penicillins and fluoroquinolones were used in 10.2% and 9% of cases, respectively. Combination therapy with 3rd-generation cephalosporins and aminoglycosides was prescribed in 5.2% of cases, while 2nd- and 4th-generation cephalosporins were used less frequently (2.2%). Other antibiotics like fosfomycin and nitrofurantoin were prescribed in 1.7% of cases. Notably, 52.4% of empiric antibiotic prescriptions were deemed appropriate according to the national guidelines.

After the culture results, around 52.8% of patients transitioned from broad-spectrum antibiotics to more targeted definitive therapy. Regarding patient outcomes, the majority (88.5%) experienced clinical improvement, while the rest faced clinical deterioration, resulting in ICU admission or death.

### 2.6. Bivariate Analysis of Factors Associated with LOS in UTI Patients

Hospital-related factors included location (South Lebanon had longer stays than Beirut, *p* = 0.007) and department (critical care units had longer stays than internal medicine, *p* < 0.001). Physician characteristics also played a role, with specialists, attending physicians, and residents associated with longer stays compared to consultants (all *p* < 0.05). Patient demographics and comorbidities were influential, with older age (r = 0.282, *p* < 0.001), male gender (*p* = 0.039), and various conditions such as diabetes, coronary artery disease, renal disease, and cerebrovascular disease all linked to extended stays (all *p* < 0.05). The type of UTI impacted stay duration, with complicated cystitis, catheter-associated UTI, and acute bacterial prostatitis associated with longer stays compared to uncomplicated pyelonephritis (all *p* < 0.05). Specific symptoms like confusion, flank pain, and longer symptom duration (>7 days) were also associated with an increased LOS (all *p* < 0.05). Microbiological findings, particularly MDROs (*p* = 0.007), and antibiotic choices (carbapenems and fluoroquinolones as empiric therapy, *p* < 0.05) were linked to longer stays. Patient outcomes, such as clinical deterioration (*p* < 0.001), and certain vital signs and lab results (higher pulse rate, lower blood pressure, higher serum creatinine) were also associated with extended hospital stays. These findings are demonstrated in Table 4.

### 2.7. Factors Influencing LOS in Hospitalized UTI Patients

A multivariable linear regression analysis was performed to examine the factors impacting the LOS for UTI patients, and the results are illustrated in Table 5. Physician position strongly predicted stay duration, with specialists (β = 1.945, *p* < 0.001), attending physicians (β = 2.179, *p* < 0.001), and residents (β = 2.236, *p* < 0.001) all associated with longer stays compared to consultants. Geographically, patients in South Lebanon experienced longer stays than those in Beirut (β = 1.102, *p* = 0.001). The UTI type was significant, with uncomplicated pyelonephritis associated with shorter stays (β = −1.402, *p* = 0.007) and acute bacterial prostatitis with longer stays (β = 1.510, *p* = 0.030) compared to complicated cystitis. Patient-related factors such as cerebrovascular disease (β = 1.620, *p* = 0.002), catheter placement (β = 1.032, *p* = 0.015), flank pain (β = 1.271, *p* < 0.001), and confusion (β = 1.828, *p* < 0.001) all significantly extended hospital stays. Regarding antibiotic therapy, carbapenems (β = 0.783, *p* = 0.004) and fluoroquinolones (β = 1.360, *p* = 0.014) were associated with longer stays compared to 3rd-generation cephalosporins. Notably, inappropriately prescribed therapy (β = 0.609, *p* = 0.022) and the presence of MDROs (β = 2.250, *p* = 0.003) both led to longer stays, while de-escalation after culture results were associated with shorter stays (β = −0.567, *p* = 0.029).

- ANOVA test of model coefficients *p*-value < 0.001.
- Model summary: Adjusted R^2^ = 0.479.
- Durbin-Watson = 1.903.

## 3. Discussion

### 3.1. Factors Associated with Prolonged LOS

In this study, LOS in UTI patients was significantly influenced by age, comorbidities, UTI classification, presenting symptoms, and MDROs. Use of carbapenems (β = 0.783, *p* = 0.004) and fluoroquinolones (β = 1.360, *p* = 0.014) was associated with prolonged LOS, as was inappropriate empiric antibiotic use (β = 0.609, *p* = 0.022). In contrast, antibiotic de-escalation following culture results reduced LOS (β = −0.567, *p* = 0.029). *Escherichia coli* was the most common pathogen (61.8%), with declining susceptibility rates.

The mean LOS in this study was 5.85 ± 2.41 days, which is comparable to a previous study in Lebanon that reported a median LOS of six days for patients with UTIs caused by antibiotic-resistant *Escherichia coli*, compared to five days for those with susceptible strains [19].

LOS was longer among patients treated in hospitals in South Lebanon compared to those treated in Beirut (β = 1.102, *p* = 0.001), likely reflecting regional disparities in healthcare infrastructure [26]. Hospitals in Beirut generally have better access to diagnostics, multidisciplinary care, and discharge planning. In contrast, hospitals in South Lebanon may face delays in laboratory results, limited access to infectious disease specialists, and fewer antimicrobial stewardship programs. These systemic factors, along with differences in case complexity due to referral patterns, can contribute to longer hospital stays.

Patients admitted to critical care units had significantly longer LOS than those in internal medicine wards (*p* < 0.001), likely due to greater illness severity and need for intensive monitoring [27].

Longer LOS was also associated with the physician’s role. Patients managed by specialists (β = 1.945, *p* < 0.001), attending physicians (β = 2.179, *p* < 0.001), and residents (β = 2.236, *p* < 0.001) stayed longer than those managed by consultants. A UK study similarly found that consultant-led multidisciplinary care is linked to shorter LOS [28]. However, this likely reflects the clinical complexity of cases typically referred to specialists and senior physicians, rather than inefficiency. Experienced clinicians often manage more severe cases requiring prolonged care and complex interventions.

Consistent with prior studies from Thailand and Iran, older age was a key determinant of prolonged LOS [29]. In our cohort, elderly patients had longer hospitalizations (r = 0.282, *p* < 0.001), possibly due to age-related changes, polypharmacy, and reduced functional reserve. A UK study also confirmed that complex cases often require longer treatment durations [24]. Specific symptoms in our study, including flank pain (β = 1.271, *p* < 0.001) and confusion (β = 1.828, *p* < 0.001), were linked to increased LOS. Catheter use (β = 1.032, *p* = 0.015) and comorbidities such as diabetes, coronary artery disease, renal disease, and cerebrovascular disease were also significant contributors (all *p* < 0.05). These factors likely delayed recovery or necessitated prolonged antibiotic therapy.

Vital sign abnormalities (e.g., high pulse and low blood pressure) and lab findings (e.g., elevated serum creatinine) were also associated with prolonged stays. Compared to complicated cystitis, uncomplicated pyelonephritis was associated with shorter LOS (β = −1.402, *p* = 0.007), while acute bacterial prostatitis was linked to longer LOS (β = 1.510, *p* = 0.030). These findings support prior evidence from Dutch hospitals that disease severity is a key driver of LOS [4].

MDRO infections were a significant predictor of prolonged LOS (β = 2.250, *p* = 0.003), consistent with findings from a respiratory care ward study in Taiwan [30]. Antibiotic-related factors influenced the LOS in three ways. First, inappropriate empiric therapy extended hospitalization (β = 0.609, *p* = 0.022), echoing results from Germany, where guideline-concordant empiric therapy shortened stays by over two days [31]. Second, the use of carbapenems and fluoroquinolones led to longer stays than third-generation cephalosporins. Third, de-escalation based on culture results reduced the LOS (β = −0.567, *p* = 0.029), aligning with data showing shorter stays with early IV-to-oral switches (4.8 vs. 9.1 days; *p* < 0.001) [31].

### 3.2. Microbiological Findings

The most common isolates were *Escherichia coli* (61.8%), *Klebsiella pneumoniae* (11.9%), *Proteus mirabilis* (7.8%), and *Pseudomonas aeruginosa* (7.5%), consistent with previous national and international reports [32,33,34]. A study from South Lebanon similarly reported *E. coli* (67.1%), *K. pneumoniae* (10%), and *P. mirabilis* (3.7%) as leading uropathogens [11]. German data also confirmed *E. coli* as the primary agent in UTIs (60%) [35]. In our study, 37.5% of isolates were ESBL-producers, compared to 32.9% in South Lebanon [11].

Monitoring local antimicrobial susceptibility patterns is essential for evaluating treatment guidelines and identifying emerging resistance trends [36]. Our findings were compared with Lebanon’s national AMR surveillance report (2015–2016) covering 13 hospitals [14]. We observed declining susceptibility in *E. coli*, *K. pneumoniae*, and *P. aeruginosa* to imipenem (current: 93.3%, 90.7%, and 66.7%, respectively). Resistance to ciprofloxacin increased significantly, particularly in *E. coli* (from 57% to 42.2%) and *K. pneumoniae* (from 71% to 51.2%). Amikacin and ceftriaxone susceptibility also declined.

These trends align with global patterns. The WHO’s Global Antimicrobial Resistance Surveillance System (GLASS) report (2022) revealed a worrying decline in carbapenem susceptibility among *Klebsiella pneumoniae*, with resistance rates exceeding 50% in some countries of the Eastern Mediterranean and South-East Asia regions [37]. EARS-Net (2022) also reported increasing third-generation cephalosporin resistance among *E. coli* isolates, with average susceptibility dropping below 50% in several European countries [38]. Similar findings exist in China with ciprofloxacin resistance rates of 53.2% in *E. coli* and 38.3% in *K. pneumoniae* [39] and persistent resistance to fluoroquinolones and aminoglycosides have been noted in US data [40].

The growing AMR burden underscores the urgent need for stewardship. Resistance increases patient morbidity, mortality, and healthcare costs, particularly in resource-limited settings like Lebanon [30]. Contributing factors include inappropriate prescribing, short antibiotic courses, and over-the-counter availability [41,42]. The development of new antibiotics remains slow due to regulatory and economic barriers, while global interconnectedness facilitates the spread of resistant strains [41].

Improving UTI management and addressing AMR in Lebanon requires strengthened adherence to national guidelines, supported by the consistent use of evidence-based antibiotic protocols. The establishment of ASPs across hospitals is essential to optimize prescribing and promote early de-escalation. Enhancing infection control practices, particularly in high-risk units, can help curb MDRO transmission. A centralized national surveillance system is also needed to monitor resistance trends and guide policy. Additionally, ongoing education for healthcare providers and public awareness initiatives are critical to promoting responsible antibiotic use.

### 3.3. Economic and Healthcare Implications

UTIs caused by resistant bacteria are associated with higher costs and a longer LOS. Infections with resistant *E. coli* increase hospitalization costs by 29% and extend LOS by one day [19]. Healthcare-associated infections with resistant pathogens result in an additional 2.69 hospital days and USD 1,807 in extra costs per patient [19]. These outcomes also lead to lost productivity and broader socioeconomic impacts. Effective infection prevention and control (IPC) measures can mitigate these effects, with higher IPC scores linked to a reduced LOS and lower healthcare costs [16].

### 3.4. Study Significance and Limitations

This study represents a significant contribution to the existing literature on urinary tract infections in Lebanon. It is the first nationwide investigation to comprehensively assess both the clinical and microbiological factors associated with prolonged LOS in hospitalized UTI patients. By combining clinical, demographic, microbiological, and antimicrobial susceptibility data, the study offers a multidimensional perspective on the drivers of hospitalization duration, particularly in the context of rising AMR. The inclusion of patients from multiple geographic regions enhances the representativeness of the findings and provides insights into regional disparities in healthcare delivery and resource allocation. Furthermore, the integration of microbiological trends with treatment practices allows for an evidence-based evaluation of current prescribing patterns and their outcomes. These findings can inform hospital policies, stewardship interventions, and public health strategies aimed at optimizing care and minimizing unnecessary hospital utilization.

The use of multivariable regression analysis strengthens the study’s analytical rigor, allowing for the adjustment of key confounding variables. Additionally, the relatively large sample size and inclusion of diverse healthcare settings improve the external validity of the results and support their applicability to similar low- and middle-income countries facing parallel challenges in infection control and antibiotic stewardship.

Despite these strengths, several limitations must be acknowledged. First, the retrospective observational design inherently limits causal inference and is subject to information bias. Patient records may have contained incomplete, inconsistent, or undocumented clinical details, particularly in regard to symptom onset timing, medication adherence, or prior outpatient antibiotic exposure, all of which may have influenced LOS.

Second, although hospitals across different Lebanese regions were included, facilities from the Beqaa region were excluded. This omission may have reduced the generalizability of findings, as healthcare delivery and prescribing patterns in the Beqaa may differ slightly from those in urban and southern regions. However, previous national studies have consistently demonstrated high rates of inappropriate antibiotic use across all Lebanese regions, including the Beqaa [43,44,45], supporting the relevance of the current findings in the broader national context.

Third, potential residual confounding remains a concern. While multivariate models adjusted for key clinical and demographic variables, certain unmeasured factors—such as clinician adherence to local or international treatment guidelines, institutional prescribing culture, hospital bed capacity, nurse-to-patient ratios, and the presence or absence of formal antimicrobial stewardship programs—may have also influenced LOS and antibiotic decision-making. Similarly, variations in physician experience, specialization, and clinical judgment, which are difficult to quantify retrospectively, could have introduced additional bias.

Fourth, standardized severity-of-illness scores such as SOFA, APACHE II, or the Pitt bacteremia score were not utilized due to the limitations of retrospective data collection. However, the study accounted for many individual components of these scoring systems, including vital signs, renal function, and the presence of comorbidities. These proxies were integrated into the analysis to approximate clinical severity and minimize confounding.

Lastly, microbiological testing was limited to hospital-acquired data and did not include community-level surveillance, which could provide a more comprehensive picture of resistance patterns in outpatient settings. Additionally, molecular analysis of resistance mechanisms (e.g., detection of ESBL or carbapenemase genes) was not performed, which could have added valuable insight into local epidemiology and transmission dynamics.

Despite these limitations, this study addresses a critical evidence gap by offering the first national-level dataset on UTI-related hospitalization and resistance in Lebanon. Its findings lay a strong foundation for future prospective research and contribute meaningfully to regional AMR monitoring efforts. This study also underscores the need for integrated, multisectoral approaches to optimize UTI management, control resistance spread, and enhance healthcare quality and efficiency in Lebanon and comparable healthcare systems.

## 4. Materials and Methods

### 4.1. Study Design and Setting

This retrospective cohort study included adult hospitalized patients with UTIs enrolled from five university hospitals located in various districts of Lebanon: two in Beirut and one each in South, North, and Mount Lebanon.

### 4.2. Study Population

Male and female patients aged 18 years or older who were hospitalized and treated for UTIs were included in this study. The exclusion criteria were applied to ensure a focused and standardized assessment of LOS for UTI patients. Outpatients were excluded because their treatment settings differ significantly from those of hospitalized patients. Individuals with multiple concurrent infections were excluded to isolate the impact of UTIs on LOS without confounding by other infections. Pregnant women and children were excluded because their treatment protocols are not addressed in the national guidelines, making it difficult to standardize their care for this analysis. Immunocompromised patients were also excluded to avoid skewing the data, as they often experience more complex and prolonged hospital courses unrelated to typical UTI management. Additionally, patients who died upon admission (i.e., those who were deceased within the first 24 h of hospitalization) were excluded from the analysis of LOS. Only those who survived beyond the first day of hospitalization were included in the calculation of LOS. These exclusions ensured that the study population reflected a more homogeneous group of immunocompetent adults with UTIs, allowing for clearer analysis of factors influencing LOS and preventing early mortality from distorting the results.

### 4.3. Sources of Data

Patients’ medical records were examined for eligibility requirements during the data collection period, which ran from 1 March 2022 to 31 December 2023. The medical records of the participants were gathered using the ICD-10 code N39 from the hospital records. Data included clinical and demographic traits of patients, signs and symptoms, type of UTIs, comorbidities, laboratory results, urine cultures, and antibiograms according to the Clinical and Laboratory Standards Institute (CLSI). The recorded data also involved the antibiotic prescriptions, dosage regimens, hospital stays, and clinical results. Patient records with incomplete key variables related to antibiotic therapy, microbiological findings, or LOS were excluded during the data-cleaning stage. Moreover, patient confidentiality was maintained during the data retrieval process. Access was restricted to the principal investigator, and all data were anonymized to remove identifiable information prior to analysis. Secure protocols were implemented for data storage and transmission, adhering to privacy laws and institutional guidelines. These measures ensured patient privacy while facilitating the necessary insights from the medical information for our research.

### 4.4. Study Size

The sample size was calculated using the following formula for cross-sectional studies: n = Z2 P (1 − P)/d2 [46], where n is the sample size, Z is the Z-value for the 95% confidence interval (which was 1.96 when α = 0.05), P is the estimated prevalence of inappropriate antibiotic prescribing based on previous study (which was 65%) [43], and d is the margin of error (which was 5%). The required sample size calculated was 349. A total of 600 medical files were initially screened in the current review, of which 199 were excluded (60 were children, 30 were pregnant females, 80 had immunocompromising conditions, and 29 had multiple infections). Therefore, after reviewing the participants’ eligibility, a total of 401 participants were enrolled in the present study.

### 4.5. Operational Definitions

1. Immunocompromised patients: patients with HIV, absolute neutrophil or total WBC count < 500/mm^3^, receiving chemotherapy, history of solid organ or hematopoietic stem cell transplant, or receiving >20 mg/day of prednisone or equivalent for more than two weeks [47].
2. Extended-spectrum-beta-lactamases (ESBLs): enzymes produced by certain bacteria, which confer resistance to a broad range of beta-lactam antibiotics, such as penicillins; first-, second-, and third-generation cephalosporins; and aztreonam (but not carbapenems) by hydrolysis of these antibiotics, and which are inhibited by β-lactamase inhibitors, such as clavulanic acid [48].
3. Multi-drug-resistant organisms (MDROs): organisms that show resistance to at least one agent in three or more antimicrobial categories [49].
4. Systemic inflammatory response syndrome (SIRS): a clinical syndrome characterized by a systemic inflammatory response to a variety of severe clinical insults, including infection, trauma, or other inflammatory conditions. SIRS is defined by the presence of at least two of the following criteria [50]:
  - Temperature: fever (body temperature > 38 °C or 100.4 °F) or hypothermia (body temperature < 36 °C or 96.8 °F).
  - Heart rate: tachycardia (heart rate > 90 beats per minute).
  - Respiratory rate: tachypnea (respiratory rate > 20 breaths per minute) or arterial carbon dioxide tension (PaCO_2_) < 32 mmHg.
  - White blood cell count: leukocytosis (white blood cell count > 12,000 cells/mm^3^), leukopenia (white blood cell count < 4000 cells/mm^3^), or the presence of >10% immature neutrophils (band forms).
5. Complicated UTI: the presence of any of the following features: functional or anatomical abnormality of the urinary tract, pregnancy, old age, diabetes mellitus, immunosuppression, urinary tract instrumentation or surgery, hospital-acquired infection, presence of a urolithiasis, symptoms for more than seven days at presentation, renal failure, renal transplant, and an infection with a pathogen resistant to broad-spectrum antibiotics [51].
6. Empiric antibiotic appropriateness: the evaluation and comparison of prescribed empiric antibiotic regimens against the recommended treatments outlined in the established national guidelines for UTI management in Lebanon [51]. This assessment includes antibiotic selection, dosage, route of administration, and duration of therapy, with a regimen considered appropriate if all four parameters align with the guidelines. Additionally, CAUTIs are assessed based on hospital protocols for nosocomial pathogens and their resistance patterns.
7. Clinical improvement: resolution or marked reduction of UTI symptoms during hospitalization accompanied by clinical stabilization, normalization of vital signs, and no requirement for escalation to Intensive Care Unit (ICU) admission. Patients discharged alive with recovery or significant symptom relief were considered clinically improved.
8. Clinical deterioration: worsening of the patient’s clinical condition during hospitalization, characterized by the need for ICU admission and/or in-hospital death. This includes progression to sepsis, hemodynamic instability, or organ dysfunction requiring critical care support. Death during the same hospital admission, when related to UTI or its complications, was classified as clinical deterioration.

### 4.6. Statistical Methods

Data analysis was conducted using IBM SPSS^®^ software version 27. Descriptive statistics are presented as frequencies and percentages for categorical variables and means with standard deviations for continuous variables. The normality of continuous variables was assessed through visual inspection of histograms and by ensuring skewness and kurtosis absolute values were less than 1.96.

Bivariate analysis was initially conducted to examine associations between LOS and various independent variables. For categorical variables, the Student’s *t*-test and ANOVA were utilized to assess the relationship between the variables. Continuous variables were analyzed using Pearson’s and Spearman’s correlation tests.

Subsequently, the disjunctive cause criterion was applied to select covariates for inclusion in the multiple linear regression model [52]. The variables incorporated into the model included patient demographics, physician data, UTI symptoms and diagnoses, laboratory data, prescribed antibiotics, urine culture microbiological findings, antibiotic de-escalation following culture results, and patient outcomes. This model was developed using a backward selection process, starting with all candidate variables and iteratively removing the least significant predictors based on *p*-values, until only those variables with a statistically significant association remained.

The assumptions of multiple linear regression were checked, including normal distribution of residuals, independence of observations, homoscedasticity, and absence of multicollinearity and outliers.

The comparison of *E. coli*’s susceptibility percentage to antibiotics to the latest national study reporting resistance patterns was conducted using a Chi-square goodness-of-fit test [14]. A *p*-value of less than 0.05 was considered statistically significant for the tests performed.

## 5. Conclusions

This study revealed that LOS among patients with UTIs is significantly influenced by a combination of physician-related, patient-specific, clinical, and microbiological factors. Notably, the physician’s position emerged as a strong predictor of LOS, with patients treated by specialists, attending physicians, and residents experiencing significantly longer stays compared to those managed by consultants, potentially reflecting varying levels of clinical decision-making or case complexity.

Importantly, the use of carbapenems and fluoroquinolones as empiric therapy, rather than guideline-recommended regimens, was independently associated with increased LOS, suggesting potential overuse of broad-spectrum antibiotics and delayed optimization of treatment. In contrast, de-escalation of antibiotic therapy following culture results was significantly associated with reduced LOS, reinforcing the value of timely, targeted antimicrobial adjustments. Furthermore, infections caused by MDROs were a key microbiological predictor of prolonged hospitalization, further emphasizing the clinical and logistical burden of antimicrobial resistance.

Together, these findings underscore the importance of evidence-based prescribing, early risk identification, and ASPs in reducing unnecessary hospital days and improving patient outcomes in the management of UTIs. Future research should focus on multicenter prospective studies to validate these findings and assess the impact of ASP interventions on LOS.

## Figures and Tables

**Table 1:** Comprehensive analysis of UTIs: types, diagnostic tests, symptoms, vital signs, and laboratory findings in the study sample.

	Frequency (Total = 401)	Percentage %
**Type of UTI**		
Pyelonephritis	190	47.4
Uncomplicated pyelonephritis	31	7.7
Complicated pyelonephritis	159	39.7
Complicated cystitis	171	42.6
Catheter-associated UTI	21	5.2
Acute bacterial prostatitis	19	4.7
**Symptoms**		
Fever/chills	205	51.1
Flank pain	160	40
Dysuria	189	47.1
Frequency/urgency	170	42.4
Nausea/vomiting	97	24.2
Burning urination	64	16
Hematuria	64	16
Lower abdominal pain	132	32.9
Confusion	45	11.2
**Symptom duration**		
Less than 7 days	254	63.3
More than 7 days	147	36.7
**Diagnostic tests used**		
Urine analysis	401	100
Urine culture	349	87
Blood culture	186	46.4
Ultrasound	71	17.7
CT scan	80	20
**Presence of systemic inflammatory response syndrome (SIRS)**	154	38.4
**Vital signs and other laboratory findings**	**Mean ± standard deviation**	
Temperature °C	37.81 ± 0.94	
Heart rate (beats/minute)	80.07 ± 15.56	
Respiratory rate (breaths/minute)	18.49 ± 3.91	
Systolic blood pressure (SBP) mm Hg	130.31 ± 22.51	
Diastolic blood pressure (DBP) mm Hg	84.43 ± 12.87	
WBC ×10^3^ cells/microliter	12.19 ± 5.09	
Creatinine (mg/dL)	1.55 ± 4.79	
**Length of hospital stay in days**	5.85 ± 2.41	

**Table 2:** Microbiological findings in cultures from hospitalized UTI patients.

Cultures Findings	Frequency	Percentage %
Positive urine culture (out of 349)	307	88
Positive blood culture (out of 186)	54	29
**Microorganisms in the cultures (out of 361)**		
*Escherichia coli*	223	61.8
*Klebsiella pnuemoniae*	43	11.9
*Proteus mirabilis*	28	7.8
*Pseudomonas aeruginosa*	27	7.5
*Enterococcus* spp.	12	3.3
*Staphylococcus coagulase-negative* *(Staphylococcus saprophyticus)*	11	3
*Acinetobacter baumannii*	4	1.1
*Candida albicans*	6	1.7
*Serratia marcescens*	3	0.8
*Stenotrophomonas maltophilia*	3	0.8
*Staphylococcus aureus (MRSA)*	1	0.3
Extended-spectrum-beta-lactamases (ESBLs)	136	37.7
Multi-drug-resistant organisms (MDROs)	23	6.4

**Table 3:** Antibiotic susceptibility rates of major bacteria isolated from UTI patients’ cultures.

Susceptibility of Main Bacteria in UTI Patients’ Cultures: Frequency (%)							
Antibiotic	*E. coli* (Total = 223)	*p*-Value	*Klebsiella pneumoniae* (Total = 43)	*Pseudomonas aeruginosa* (Total = 27)	*Other Enterobacterales* (Total = 31)	*Enterococcus* spp. (Total = 12)	*Coagulase-negative Staphylococcus* spp. (Total = 11)
Amikacin	195 (87.4%)	0.191	37 (86%)	22 (81.5%)	29 (93.5%)		
Amoxicillin/clavulanic acid	51 (22.9%)	<0.001 *	20 (46.5%)		18 (58.1%)		6 (54.5%)
Ampicillin	28 (12.6%)	<0.001 *	10 (23.3%)		11 (35.5%)		
Aztreonam	125 (56.1%)	0.856	28 (65.1%)	18 (66.7%)	27 (87.1%)		
Cefepime	116 (52%)	0.005 *	30 (69.8%)	20 (74.1%)	26 (83.9%)		
Cefoxitin	83 (37.2%)	<0.001 *	22 (51.2%)		19 (61.3%)		
Ceftazidime	98 (43.9%)	<0.001 *	27 (62.8%)	19 (70.4%)	26 (83.9%)		
Ceftriaxone	100 (44.8%)	<0.001 *	26 (60.5%)		25 (80.6%)		9 (81.8%)
Cefuroxime	72 (32.3%)	<0.001 *	21 (48.8%)		18 (58.1)		
Ciprofloxacin	94 (42.2%)	<0.001 *	22 (51.2%)	15 (55.6%)	21 (67.7%)		
Gentamicin	155 (69.5%)	0.359	29 (67.4%)	21 (77.8%)	28 (90.3%)		
Imipenem	208 (93.3%)	0.001 *	39 (90.7%)	18 (66.7%)	30 (96.8%)		
Nitrofurantoin	161 (72.2%)	<0.001 *	19 (44.2%)		17 (54.8%)		
Piperacillin/ tazobactam	165 (74%)	0.508	31 (72.1%)	17 (63%)	28 (90.3%)		
Tigecycline	210 (94.2%)	0.170	41 (95.3%)		31 (100%)	11 (91.7%)	11 (100%)
Trimethoprim/ sulfamethoxazole	90 (40.4%)	<0.001 *	17 (39.5%)		16 (51.6%)		10 (90.9%)
Ampicillin						9 (75%)	
Vancomycin						10 (83.3%)	11 (100%)
Clindamycin							10 (90.9%)

* Denotes a significant *p*-value compared to national data.

**Table 4:** Bivariate analysis of the factors associated with length of hospital stay for UTI patients.

	Mean ± SD or Correlation Coefficient	*p*-Value	95% Confidence Interval	
			Lower	Upper
**Hospital area**		<0.001 *		
Beirut (reference)	5.50 ± 2.31			
North Lebanon	5.19 ± 2.43	0.929	−5.75	1.19
South Lebanon	6.48 ± 2.37	0.007 *	−1.77	−1.93
Mount Lebanon	6.19 ± 2.33	0.196	−1.64	0.27
**Hospital department**				
Internal medicine	5.64 ± 2.32	<0.001 *	−1.69	−0.50
Critical care units (ICU/CCU)	6.74 ± 2.57			
**Physician position**		0.004 *		
Consultant (reference)	4.70 ± 1.37			
Specialist	5.85 ± 2.51	<0.001 *	−1.87	−0.43
Attending	6.23 ± 2.42	<0.001 *	−2.37	−0.68
Resident	6.11 ± 2.38	0.015 *	−2.61	−0.19
**Physician specialty**		0.010 *		
Infectious diseases (reference)	5.60 ± 2.17			
Internal medicine	6.06 ± 2.71	1.00	−1.31	0.40
Critical care	6.97 ± 2.57	0.009 *	−2.53	−0.21
Urology/nephrology	5.42 ± 2.39	1.00	−0.93	1.29
Others (emergency medicine/general surgery)	6.04 ± 2.42	1.00	−1.86	0.98
**Physician gender**				
Male	5.85 ± 2.51	0.926	−0.48	0.52
Female	5.82 ± 2.03			
**Patient age**	0.282	<0.001 *	0.18	0.37
**Patient gender**				
Male	6.16 ± 2.44	0.039 *	0.03	0.99
Female	5.65 ± 2.37			
**Marital status**		0.358		
Single (reference)	5.60 ± 2.42			
Married	5.87 ± 2.43	1.00	−0.98	0.46
Divorced/widow/widower	6.30 ± 2.19	0.474	−1.89	0.49
**Medical coverage**				
No	5.73 ± 2.25	0.410	−0.68	0.28
Yes	5.93 ± 2.51			
**Smoking status**				
Not a current smoker	5.78 ± 2.34	0.465	−0.68	0.31
Current smoker	5.97 ± 2.54			
**Patients’ comorbid conditions**				
Diabetes				
No	5.66 ± 2.43	0.034 *	−1.02	−0.04
Yes	6.19 ± 2.33			
Hypertension				
No	5.65 ± 2.42	0.145	−0.83	0.12
Yes	6.01 ± 2.38			
Dyslipidemia				
No	5.69 ± 2.43	0.455	−0.36	0.81
Yes	5.87 ± 2.29			
Congestive heart failure				
No	5.79 ± 2.41	0.141	−1.41	0.20
Yes	6.39 ± 2.36			
Coronary artery disease				
No	5.72 ± 2.39	0.037 *	−1.22	−0.39
Yes	6.35 ± 2.38			
Renal disease				
No	5.74 ± 2.35	0.010 *	−1.76	−0.25
Yes	6.74 ± 2.68			
Neurological disorders				
No	5.66 ± 2.37	0.002 *	−1.89	−0.44
Yes	6.83 ± 2.57			
Arrhythmias				
No	5.85 ± 2.41	0.798	−0.84	1.08
Yes	5.73 ± 2.37			
Urolithiasis				
No	5.41 ± 2.40	0.172	−0.22	1.22
Yes	5.90 ± 2.41			
Catheter placement				
No	5.61 ± 2.42	<0.001 *	−1.48	−0.41
Yes	6.55 ± 2.21			
Benign prostatic hypertrophy				
No	5.81 ± 2.40	0.229	−1.64	0.39
Yes	6.43 ± 2.46			
**Antibiotic allergy**				
No	5.87 ± 2.39	0.207	−0.54	2.48
Yes	4.90 ± 2.76			
Antibiotic use within 3 months				
No	5.86 ± 2.38	0.598	−0.59	1.03
Yes	5.64 ± 2.59			
**Type of UTI**		0.003 *		
Uncomplicated pyelonephritis (reference)	4.54 ± 2.03			
Complicated pyelonephritis	5.75 ± 2.34	0.099	−2.52	0.11
Complicated cystitis	5.95 ± 2.36	0.026 *	−2.71	−0.09
Catheter-associated UTI	6.81 ± 2.56	0.008 *	−4.15	−0.37
Acute bacterial prostatitis	6.74 ± 2.90	0.017 *	−4.13	−0.24
**Patients’ symptoms**				
Flank pain				
No	5.85 ± 2.42	0.033 *	−0.82	−0.12
Yes	6.83 ± 2.37			
Dysuria				
No	5.64 ± 2.29	0.122	−0.08	0.85
Yes	6.02 ± 2.49			
Hematuria				
No	5.68 ± 2.36	0.561	−0.45	0.83
Yes	5.87 ± 2.41			
Fever/chills				
No	5.47 ± 2.11	0.327	−0.41	1.24
Yes	5.88 ± 2.43			
Lower abdominal pain				
No	4.94 ± 2.66	0.014 *	−1.78	−0.20
Yes	5.94 ± 2.36			
Confusion				
No	5.63 ± 2.29	<0.001 *	−2.64	−1.19
Yes	7.55 ± 2.57			
Nausea/vomiting				
No	5.42 ± 2.13	0.032 *	−1.07	−0.49
Yes	5.98 ± 2.47			
Frequency/urgency				
No	5.30 ± 2.02	0.004 *	−1.22	−0.24
Yes	6.03 ± 2.49			
Burning urination				
No	5.90 ±2.45	0.321	−0.31	0.98
Yes	5.56 ± 2.14			
**Symptoms duration**				
Less than 7 days	5.65 ± 2.33	0.028 *	−1.04	−0.06
More than 7 days	6.19 ± 2.49			
**Microbiological findings**				
Extended-spectrum-beta-lactamases (ESBLs)				
No	5.94 ± 2.31	0.072	−1.07	0.05
Yes	6.46 ± 2.57			
Multi-drug-resistant organisms (MDROs)				
No	6.04 ± 2.39	0.007 *	−2.65	−0.42
Yes	7.57 ± 2.43			
**Microorganisms in culture**		0.197		
*Escherichia coli* (reference)	6.04 ± 2.38			
*Klebsiella pneumoniae*	5.97 ± 2.55	1.00	−1.32	1.45
*Proteus mirabilis/Serratia marcescens*	6.50 ± 2.93	1.00	−2.10	1.17
*Pseudomonas aeruginosa*	6.95 ± 1.98	1.00	−2.69	0.86
*Enterococcus species*	6.00 ± 1.85	1.00	−2.69	2.77
*Staphylococcus coagulase-negative (Staphylococcus saprophyticus)*	4.85 ± 2.34	1.00	−1.73	4.10
*Acinetobacter baumannii*	9.00 ± 2.64	0.994	−7.37	1.45
Others *(Candida albicans/Stenotrophomonas maltophilia/Staph aureus)*	6.80 ± 2.16	1.00	−4.20	2.67
**Empiric antibiotic**		0.021 *		
Third-generation cephalosporins (reference)	5.44 ± 2.29			
Carbapenems	6.32 ± 2.32	0.037 *	−1.77	−0.01
Fluoroquinolones	6.14 ± 2.87	0.048 *	−1.69	−0.09
Penicillin derivatives	5.85 ± 2.40	1.00	−1.74	0.92
Combination of third-generation cephalosporins and aminoglycosides	5.24 ± 2.04	1.00	−1.53	1.93
Other cephalosporins (second- and fourth-generation)	5.77 ± 3.15	1.00	−2.85	2.18
Others (fosfomycin and nitrofurantoin)	5.71 ± 1.70	1.00	−3.11	2.56
**Appropriateness of empiric antibiotic**				
Appropriate	5.64 ± 2.31	0.106	−0.08	0.86
Inappropriate	6.03 ± 2.48			
**Outcome of therapy**				
Clinical improvement	5.83 ± 2.37	<0.001 *	0.64	1.51
Clinical deterioration (ICU admission/death)	6.93 ± 2.67			
**Vital signs and other lab tests**				
Temperature °C	0.180	0.108	0.117	0.258
Respiratory rate (breaths/minute)	0.075	0.136	−0.024	0.171
Pulse (beats/minute)	0.157	0.002 *	0.053	0.250
SBP mm Hg	−0.101	0.042 *	−0.197	−0.004
DBP mm Hg	−0.148	0.018 *	−0.243	−0.051
WBC × 10^3^ cells/µL	0.062	0.212	−0.036	0.159
Serum creatinine (mg/dL)	0.112	0.025 *	0.014	0.208
**Systemic inflammatory response syndrome (SIRS)**				
No	5.71 ± 2.38	0.142	−0.85	0.12
Yes	6.07 ± 2.44			

* Denotes a significant *p*-value (<0.05).

**Table 5:** Factors affecting LOS in hospitalized UTI patients—multivariable linear regression analysis.

Factor	Unstandardized β	Standardized β	*p*-Value	95% Confidence Interval		VIF
Physician gender (reference: male)	−0.594	−0.104	0.053	−1.197	0.008	1.123
**Physician Position: (reference: consultant)**						
Specialist	1.945	0.400	<0.001 *	1.051	2.839	3.411
Attending	2.179	0.379	<0.001 *	1.133	3.224	3.345
Resident	2.236	0.268	<0.001 *	1.098	3.373	1.871
**Hospital Area: (reference: Beirut)**						
South Lebanon	1.102	0.212	0.001 *	0.476	1.728	1.467
Mount Lebanon	0.789	0.107	0.079	−0.093	1.671	1.438
**Type of UTI (reference: complicated cystitis)**						
Uncomplicated pyelonephritis	−1.402	−0.144	0.007 *	−2.410	−0.394	1.110
Acute bacterial prostatitis	1.510	0.112	0.030 *	0.147	2.873	1.091
**Patient-related factors**						
Neurological disorders	0.701	0.107	0.044 *	0.0180	1.383	1.144
Catheter placement	1.032	0.127	0.015 *	0.200	1.864	1.064
Flank pain	1.271	0.249	<0.001 *	0.605	1.937	1.720
Confusion	1.828	0.258	<0.001 *	1.081	2.576	1.127
**Empiric Antibiotic Therapy (reference: 3rd-generation cephalosporins)**						
Carbapenems	0.783	0.161	0.004 *	0.255	1.311	1.188
Fluoroquinolones	1.360	0.133	0.014 *	0.281	2.439	1.122
Appropriateness of prescribed therapy (reference: appropriate)	0.609	0.125	0.022 *	0.187	1.131	1.156
De-escalation after culture	−0.567	−0.117	0.029 *	−1.077	−0.057	1.231
Multidrug-resistant organism	2.250	0.175	0.003 *	0.634	3.046	1.365
Patient outcome (reference: clinical improvement)	0.776	0.106	0.050	−0.010	1.552	1.139

* Denotes a significant *p*-value < 0.05.

## Data Availability

The original contributions presented in this study are included in the article. Further inquiries can be directed to the corresponding author.

## Abbreviations

The following abbreviations are used in this manuscript:

AMR	Antimicrobial resistance
ASPs	Antimicrobial stewardship programs
ESBL	Extended-spectrum-beta-lactamase
LOS	Length of stay in hospital
MDROs	Multidrug-resistant organisms
SIRS	Systemic inflammatory response syndrome
UTI	Urinary tract infection
